# A simple demonstration of a privacy-preserving de-centralised genotype imputation workflow

**DOI:** 10.1371/journal.pone.0353078

**Published:** 2026-08-20

**Authors:** Alban Letaillandier, David Picard-Druet, Thomas E. Ludwig, Gaëlle Marenne, Anthony F. Herzig

**Affiliations:** 1 Inserm, Univ Brest, EFS, UMR 1078, GGB, Brest, France; 2 CHU Brest, Brest, France; Jaypee University of Information Technology, INDIA

## Abstract

Recently, a number of studies have looked at the problem of privacy and data-sharing restrictions in the context of missing-genotype imputation servers. This relates to the most typical imputation pipelines which involve a whole-genome sequenced haplotype reference panel being compared to genotyped study individuals (who have missing data to be imputed). Hence, involving two datasets from separate sources coming together in one informatic environment, where relatively complicated statistical models are applied: specifically, hidden Markov modelling. We give a short review of the current literature in this domain, observing three prevalent strategies: complicated data encryption, technical solutions to secure computation environments, and rearrangements of haplotype data to provide anonymisation. We embarked on a thought experiment to provide a potential fourth type of solution involving federating the different internal tasks within the statistical methods used for imputation. This idea is relevant considering there is currently motivation for federated analyses platforms in Europe for making combined inference across multiple genomic data resources. Our solution allows for very simple manipulations to protect sensitive individual level data, which enable imputation algorithms to complete on simple plain-text files. We provide here an illustration of how such a federated imputation server could be put in place, along with associated code, including a simple implementation of the Li-Stephens haplotype mosaic model to achieve the imputation of missing genotypes. We name our general framework ANONYMP for anonymised imputation. A demonstration of the concept is given involving simulated data generated with msprime. We show that dividing different parts of the required calculations for statistical imputation between several sites is a valuable new avenue in the field of privacy-preserving imputation server development.

## Introduction

Despite the tumbling costs of whole-genome sequencing (WGS), the tried and tested strategy of array genotyping followed by haplotyping and missing genotype imputation still finds its place in modern genetic epidemiology and population genetics study designs [1,2]. WGS datasets come with multiple burdens: cost, data generation, storage and computation capacity, and bioinformatic expertise. Imputation remains an attractive study design for many reasons. Firstly, the increasing number of human genomes that have been whole-genome sequenced has led to a greater number or increasingly large and diverse imputation panels being created [3–5]. There has also been an increase in the creation and availability of local reference panels [6–12]: panels of WGS datasets specific to different region of the global genetic landscape in human populations, often with a recruitment coming from a specific geographic region, cultural group but in most cases from a specific nation. Secondly, there is an increasing possibility to replace array-genotyping with low-coverage WGS [13–15]. This technique gives added benefits for imputation of individuals from parts of the ancestry spectrum for whom past choices of known single-nucleotide polymorphisms (SNPs) for genotyping arrays are less pertinent for facilitating haplotype-matching. For the most part, genotyping arrays have been designed to capture population structure in European-ancestry populations [16,17].

An important consideration of imputation studies is that by nature they require two datasets to work in harmony, these we will refer to as the reference panel and the target panel. The reference panel being the phased haplotypes from WGS for the reference individuals who are used for imputation and the target panel being the individuals with missing data, requiring imputation, with array or low-coverage WGS data. In this work we only consider the scenario of array data for the target panel. In many circumstances, the two datasets are generated separately by different teams of researchers. Setting aside the multitude of potential difficulties in combing population genetic datasets without introducing any bias through batch effects, there is the added logistical challenge of finding an environment for the imputation to take place in a way such that the two owners of the two datasets remain within whatever regulatory and ethical constraints pertain to their data regarding the privacy of the participating individuals [18,19]. Typically, a haplotype reference panel represents a highly complex, versatile, and detailed resource; and hence a precious one. The combination of containing thousands of whole-genome sequencing samples (containing many rare genetic variants), having assigned population labels, and often with sample overlap with cohorts used in genome-wide association studies (GWAS), has unsurprisingly led to careful restrictions on the use of such imputation panels. While certain panels are freely available, such as the combined call-set 1000G+HGDP [5] comprising the 1000 Genomes Project [20] individuals with those of the Human Genetic Diversity Panel [21,22], increasingly often they are only accessible via designated imputation servers, or require data-transfer agreements to be put in place specifically mentioning that such panels are only to be used for the purposes of imputation [4,23].

For many years, two possible imputation servers existed, one at the Sanger institute [4] and the other at the University of Michigan [24]. It is at the Michigan server that the largest and generally regarded as most complete and highly performing imputation panel TOPMED [3] is available. Many other imputation servers have recently gone online [25–30]. The idea of an imputation server is that it provides a safe environment for the reference panel to be housed and to avoid divulging the full dataset to each researcher wishing to perform imputation. The fact that further imputation servers have gone online in other parts of the world, may in part be a response to the problem that researchers may have certain restrictions preventing them from sending their data to overseas imputation servers. An example that the authors here are most familiar with being that GDPR (https://gdpr.eu/) prevents researcher in Europe sending out individual genetic data to the United States of America and hence the use of imputation servers outside of the EU is technically not permitted; and indeed the same issue would exist in the opposite direction due to HIPAA [31] guidelines in the United States of America. Even transfers between EU nations require considerable administrative framework to ensure that GDPR regulations (which can have different interpretations in different locations) are correctly respected at both ends. In order to achieve compliance, it is strategic to be able to demonstrate that sensitive data is being exposed to the minimum of risk necessary in order to achieve the aim of the analysis [32].

Such concerns are not specific to the EU, there could be many circumstances where the two actors involved (the holder of the reference panel and the holder of the target panel) would have strict restrictions on the use and sharing of their datasets. This leads to a recent and niche research area of privacy-preserving methods for imputation servers, of which we give a short overview here. Our work serves as a commentary, but we will also put forward potential additional methodological solutions to the logistical restriction of imputation servers.

A previous short review of privacy concerns and imputation was put forward by Sherman [33] which outlines the background of genotype imputation and imputation servers, current considerations regarding data-sharing that impact the potential usage of imputation servers and early work in the domain of privacy-preserving imputation. Three central themes appear in the current literature: 1) using complicated (often homomorphic) encryption methods to protect the data, 2) relying on a trusted secure third-party environment, and 3) manipulating reference panels to create ‘synthetic’ data such that imputation can still be achieved without directly using complete individual-level haplotypes.

Kim et al. [34], Sakar et al. [35], and Gürsoy et al. [36] all approach the problem by proposing homomorphic encryption based calculations to achieve the imputation. Here, both reference and target data are safely encrypted and can be analysed together without de-encryption. The downside of this approach is an increase in computational burden coming from calculation on encrypted data and that neither method performs hidden Markov modelling of haplotype mosaics [37] for the imputation and hence have less precision than leading methods [24,38,39]. Homomorphic encryption also greatly increases the volume of data that may need to be transferred. Full implementations of Hidden Markov models (HMMs) with homomorphic encryption may currently be in development [40], but are yet to be deployed at the time of writing. Dokmai et al. [41] demonstrate the loss of accuracy entailed by homomorphic encryption approaches and put forward an application of the minimac [24] imputation software on Intel SGX’s Trusted Execution Environment framework; hence providing a solution relying on a highly trustworthy third party to perform the imputation. The same group has also recently extended their methods to provide a solution for haplotype phasing [42]. Such third-party server solutions have also been put in place for applications of genome-wide association studies (GWAS) [43–45]. Cavinato et al. [46] present a simple solution of creating new haplotype mosaics from a reference panel to render it anonymized and hence sharable. This avoids sharing complete individual level data though it would likely not prevent the re-identification of individuals that have contributed to the reference panel. The approach in question is elaborated on and developed further to also protect both the reference and target panel in Zhi et al. [47]. The key idea is that genotype imputation algorithms require haplotype data but only ‘locally’; to impute missing variants in a given genomic region, individual haplotypic data is required within and near that region but not across the whole genome. Hence, breaking and recombining the haplotypes is not significantly harmful to imputation. And even if haplotypes are broken and recomposed within the region in question, if this it done in a sensible way (with a realistic recombination model), the imputation algorithm can still deal well with this. As an aside, we also point the reader to Zhi et al. [47] for a thorough literature review of the current standpoints regarding privacy-preservation in the analysis of genomic data. Finally, Mosca and Cho [48] have recently evaluated the potential for the reconstruction of private genomes in the reference panel by carefully constructing synthetic target data; they also describe potential mitigation measures to avoid such attacks. A mention should also be made to Yelmen et al. [49] who use machine learning to create entirely synthetic imputation reference panels that are thus safe to share.

To summarize, the many recent papers on the subject show the interest of developing methodological solutions to allow for greater privacy preservation in the imputation-server framework. We observe the following: homomorphic encryption is appealing in nature but may be cumbersome to put in place in terms of additional calculation costs and potential loss of precision; going beyond two parties, so that the reference panel and target panel holders retain control of their sensitive data, and a trusted third party performs the imputation would seem essential; greater data protection can come from either breaking and reforming haplotypes [46], or simply by performing imputation in relatively small regions with different sample identifiers for reference and target haplotypes.

In this work, we present a combination of these different approaches, based on the following observations: first, a federated calculation environment would add data security by spreading the different tasks involved in imputation algorithms across different nodes; second, splitting the genome into many small regions would obscure the individual level data involved in both the reference and target panels, while losing little precision; and third, that using simpler data encryption would facilitate calculations. We employ simple tactics of randomly shuffling records (genetic variants and haplotypes) and adding noise to the genetic data (permuting genotypes or adding dummy variants and haplotypes). Shuffling of haplotypes has previously been explored in Cavinato et al. [46] and in Yelmen et al. [49]. The addition of noise to genetic data to ensure privacy was demonstrated by Harary [50]. But in all of these cases some imputation accuracy may have been sacrificed; we apply shuffling and noise in a manner that doesn’t impact the final calculations; in particular all noise that we add to genetic data is completely cancelled out downstream. The shuffling of genetic variants is key to the privacy-preserving model as linkage-disequilibrium structures could otherwise be harnessed to reveal sensitive genetic data [51]. Shuffling and the inclusion of dummy data entries is an established method for establishing data-privacy [52] and has been proposed as a valuable technique for federated kinship estimation [53]. To our knowledge, it has not been applied (in the manner that we propose here) to sharing packets of genomic data for a federated imputation analysis. As described in Ye et al. [51], while SNP re-ordering is effective it is inherently problematic for analyses using HMMs. We will hence isolate the single moment of the HMM when the SNPs need to be in chromosome order but still apply random shuffles upstream and downstream to amplify privacy.

The combination of these approaches allows, in a new potential anonymised imputation framework ANONYMP that we present here, to ensure data protection. The first key idea is that the Li-Stephens [37] HMM model used for imputation only needs the agreement status between reference panel and target panel alleles for all genetic positions present in the two datasets, as well as the distances between adjacent genetic positions. However, it is not essential to know where these positions are on the genome, nor what the alleles (nucleotides) are (they just need to be seen to be the same or not). Adding some noise in the genetic distance information is not significantly harmful to the HMM model accuracy and performance and helps hide the genetic positions [47]. Furthermore, the calculation of the agreement status does not require the variants to be in the same order, hence we use a similar idea to Dervishi et al. [53] for kinship calculation. We can also add two further measures to the obscure the data at this stage, adding symmetric noise to genotypes and synthetic data records. The second key idea is that the HMMs run only on the positions in common between the two panels, meaning that only these positions need to be shared with the external trusted third parties for imputation. The third key idea is that while imputation works best with thousands of reference panel haplotypes available, typically far fewer are actually used when imputing a given target individual in a given region (see discussion of the K parameter later on in this work). The fourth key idea is that the output of the Li-Stephens HMM, posterior decoding via the forward-backward algorithm [54], does not need to be immediately used to calculate expected allele counts for missing genotypes in the target individuals, the posterior coding output can be passed (with encryption) between actors.

The inspiration for the development of ANONYMP comes in part from the Genome of Europe project (https://genomeofeurope.eu/); a plan for federated analysis of 100,000 whole-genomes across multiple EU countries. Here, there will be the desire to perform imputation where target and reference data may come from different sources and regulatory considerations imply that they cannot be shared easily. A potential solution would be a third party imputation server, with a GDPR compliant infrastructure such as in Munich [28]. However, in order to fully respect data-sharing legislation, we envisage a scenario where several actors who trust one another wish to work together to perform imputation and hence desire to set up a secure infrastructure designed to comply with strict data-sharing legislation without overly compromising on imputation quality. We therefore do not focus this work on dishonest behaviour and collusion among the operators involved, nor on external attacks on the system such as have been discussed in previous work on secure imputation servers [41].

The proposed framework remains largely a thought experiment: thus, we employ naive encryptions and a very simple application of the Li-Stephens model. Indeed, in this work we discuss encryptions in only a very loose sense. This work aims at assessing the feasibility of such a federated framework, and whether it could be relevant for future cross-border projects involving federated calculation. This is therefore an initial exploration of some new ideas into the emerging field of privacy-preserving imputation servers. To render the approach fully robust would require further considerations around the security of transmitted messages and data packages.

## Methods

We present here a secure imputation framework involving five operators. The first two are the conventional operators who provide the target data to be imputed and the reference panel. The three additional third-party ones share out the imputation algorithm steps in such a way that no single operator can access clear sensitive data. The five include: the ‘1-User’, being the operator who holds the target-data to be imputed; the ‘2-Reference’, being the operator who holds the haplotype reference panel for imputation; and three external trusted third parties that we name ‘3-Compare’, ‘4-PPM’ (for posterior probability matrix), and ‘5-Product’. In practice, the last three external operators could potentially be combined in one centre with the possibility to have three different secure calculation nodes virtually isolated in hermetic informatic environments. The essential idea is that each operator only gets the data needed to realise a specific step of the imputation process. So, if the other security measures fail to prevent a single operator being compromised, the risk of leaked sensitive data is minimized. For this proof of concept, the ANONYMP implementation doesn’t focus on imputation results or performance. Furthermore, we don’t specify how operators communicate between each other, we just postulate that each operator can’t directly access files that other operators have not shared with them.

**Fig 1:**
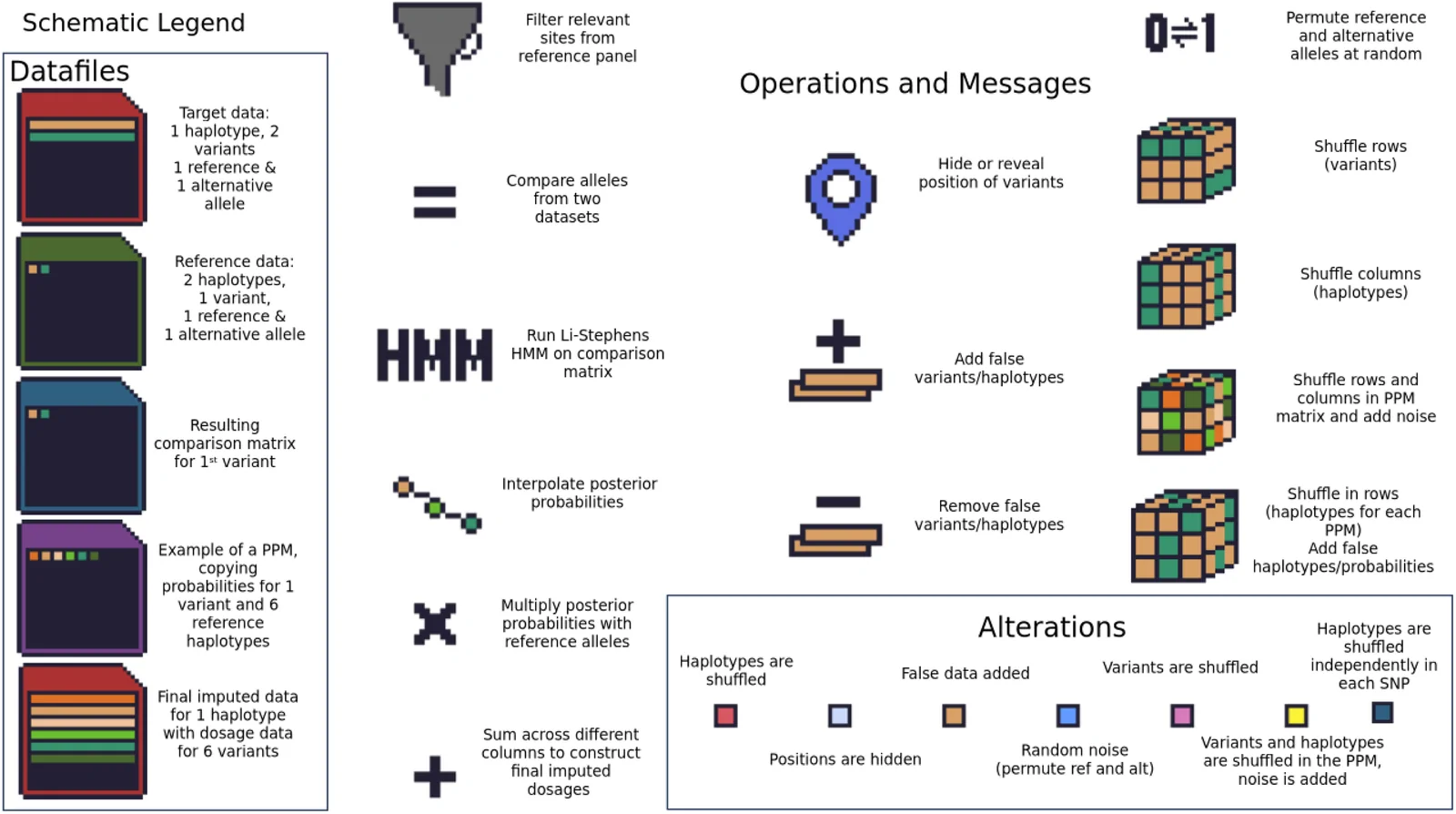
A detailed legend of the elements of the schematic in subsequent Figures (2 and 3) along with additional symbols for different types of possible simple random and reversible encryptions (Alterations) including rearranging rows and columns, removing or hiding all chromosome and position information, permuting reference and alternative alleles in individual level data, adding spurious data, and adding random noise.

Fig 1 presents a legend of symbols that will be used in subsequent figures to demonstrate the ANONYMP workflow. In Fig 1, the “Alterations” refer to the simple encryption that we apply in our framework, which are encryptions in only a loose sense, as we are simply performing some basic modifications on the datasets. More strenuous encryptions can be made on the actual files and messages send between operators, here our main focus is on the simple encryptions or modifications to the files that render them non-informative in regard to individual level genotypes but nonetheless allow for Hidden Markov modelling.

**Fig 2:**
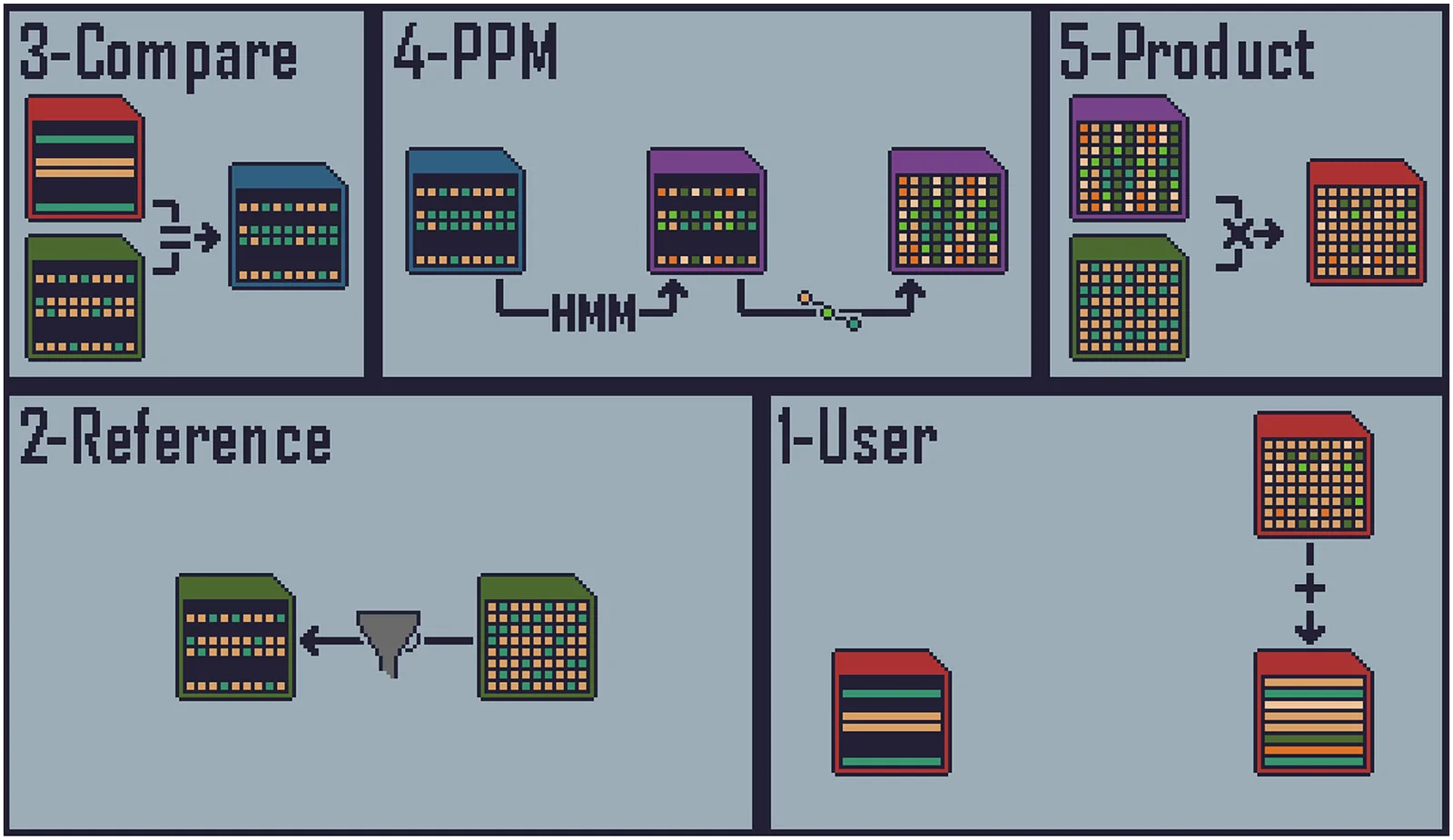
The different operators and operations are laid out. Datafiles are represented graphically with variants in rows and individuals in columns as described in Fig 1. The schema starts in the bottom left corner of the 1-User box where we see a single target individual with genotyping array positions, target data is represented as red files, from there onwards the imputation protocol proceeds clockwise. The next step is to filter the same positions from the reference panel (green files in the 2-Reference box). Then both 1-User and 2-Reference sent their data to the 3-Compare server, where a comparison matrix (blue file) is generated which simply records the agreement or not of the two datasets. Agreement here refers to a logical test of whether two haplotypes carry the same allele at a given position. The comparison matrix is passed to the 4-PPM server where the HMM is performed to produce a posterior probability matrix (purple file), followed by linear interpolation to produce posterior probabilities at sequencing positions not present on the genotyping array. These posterior probabilities are then sent to 5-Product who also receives sequencing data of reference panel haplotypes from 2-Reference. 5-Product performs the required multiplications to calculate the component parts of the expected imputed minor allele counts for the target individuals. These components are then sent from 5-Product to 1-User, who combines them to for the final result: imputed dosages for previously missing genotypes.

Fig 2 describes the (underlying) role of each operator, the operations that need to be performed are laid out and tasks are separated and allocated to the different operators. 1-User provides the target genotype data to 3-Compare and receives the final imputed genetic sequence from 5-Product at the end of the entire operation. 2-Reference provides subsets of the reference panel, firstly to 3-Compare and secondly to 5-Product. 3-Compare compares the target panel to the reference panel. It sends a comparison matrix to 4-PPM. 4-PPM computes the Posterior Probability Matrix (PPM, the output of the Li-Stephens HMM) from the comparison matrix and also perform linear interpolation of posterior probabilities. 5-Product computes the expected allele count for each SNP according to each haplotype. It does it by a simple product of the data from 2-Reference and the interpolated statistics provided by 4-PPM. This is then passed back to 1-User who sums this result over columns to form the final imputed dosages.

In Fig 2, no data security measures are added, for example 3-Compare has access to individual level data for both the target and reference panels. However, we could imagine that if the same encryption were added to both files, 3-Compare could still complete their tasks without accessing un-encrypted data. In fact, the comparison can be made with variants that are not in chromosomal order and could even involve completely spurious variants and haplotypes. The comparison also does not need any information about which chromosome or which variants are within the two files, just that they are the same set. The 4-PPM server requires this comparison matrix, that the variants are in order, that there are no spurious entries, and some (potentially approximate) idea of genetic distances between adjacent variants to perform the HMM calculations. The 5-Product operator needs to multiply linearly interpolated posterior copying probabilities with sequencing variants from the reference panel, again with no need for knowledge of chromosome position, and indeed random noise and spurious data points can be added to the calculation as long as they can be removed later.

**Fig 3:**
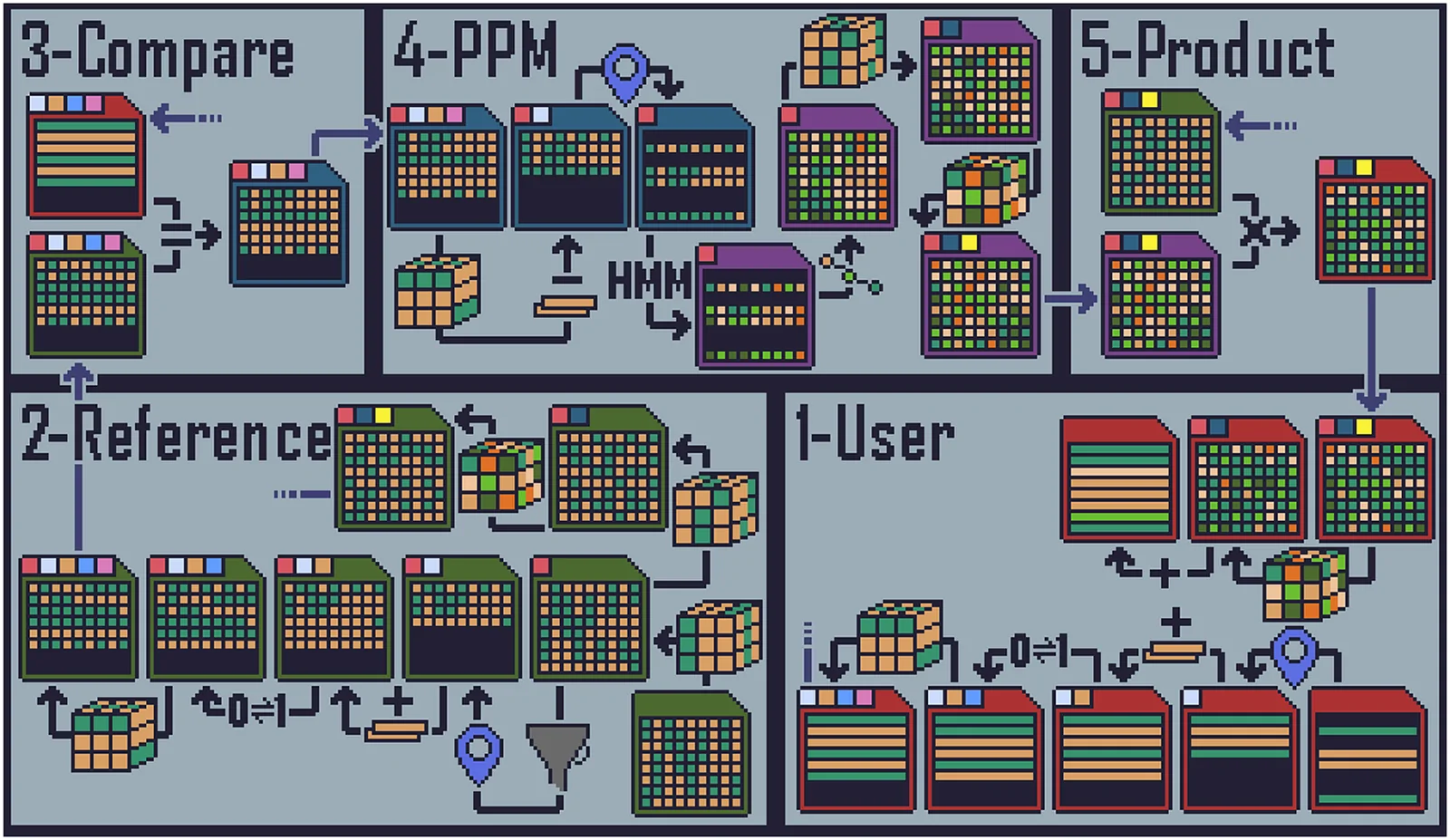
The full schematic of ANONYMP including the various data manipulations (detailed in Fig 1) to protect both reference and target data.

In Fig 3, we include the additional steps of ANONYMP to preserve privacy of both target and reference datasets. To read through the summary given in Fig 3, begin at the bottom right corner of the 1-User panel. Then move along the bottom row of the 1-User panel, where first the information about chromosome and base-pair position are hidden, and spurious data are added (in our current application, this is done in such a manner so that all of the chromosome ‘chunks’ have the same size of 2^10^ variants or rows in a ${\text{2}}^{\text{1}\text{0}}\times \text{1}\;$matrix for each 1-User haplotype to be imputed). Then, all variants are coded as REF/ALT (denoting reference or alternative alleles) so no specific allele information is retained and the REF/ALT status of all alleles in the target haplotypes are permuted randomly (each allele has a 50% chance of being inversed from REF to ALT or vice-versa), and finally the order of the rows is randomised. Messages are exchanged with 2-Reference so that equivalent operations can be performed. Hence, when 1-User and 2-Reference pass their modified files (denoted as file symbols with additional alterations represented by coloured squares on the top left corner of datafile symbols), they are encrypted in the same manner. 2-Reference makes an additional modification (bottom-right corner of the 2-Reference box); the order of the haplotypes in the reference panel are shuffled, hence the 3-Compare server does not work with datafiles with the same column order for each iteration. As all these operations are specific to each single target haplotype to be imputed, the eagle-eyed reader may spot that this would all entail that the reference panel data is transferred a very large number of times to the 3-Compare server. As this would be impractical, it would be more pragmatic if the 2-Reference operator were to send instructions to 3-Compare to re-shuffle and re-noise previously received data from previous imputation runs, in a manner avoiding ‘de-encrypting’ the reference data. This is entirely feasible as the encryptions proposed are ensured to be commutable operations and hence can be combined into a single instruction involving changing 0s to 1s and vice-versa across the reference datasets (green files) held by 3-Compare. Hence, what is given in Fig 3 corresponds to the first imputation run on the hypothetical multi-site platform. There are multiple ways to achieve the same goal, but the essential idea is that 3-Compare has two completely non-informative files but whose differences correspond to real differences between the alleles in the target and reference panel in a certain genomic region (note again that 3-Compare does not need to know where this region is).

The comparison matrix (blue files) still potentially contains spurious entries, and the variants are not in genomic order, they are hence not highly informative about the original data, but some things could potentially be inferred. A row with mostly discordant data may likely represent a variant which is likely rare, and the target individual holds the alternative allele. This could begin to become dangerously informative once the variants (rows) are out back in order which is required to run the HMM. This is why we propose to isolate this task and at this point pass to a 4^th^ operator: 4-PPM. Here, rows are put back in order and spurious entries are removed as 1-User has communicated with 4-PPM and revealed the required manipulations to do so. Even though there is no need to re-inject chromosome or position information, 4-PPM needs some estimation of recombination rates between adjacent sites, though this information can be approximative without harming the imputation accuracy, which helps to avoid re-identification of the region based on comparison to a known genetic recombination map. The idea of adding noise to the recombination map is also discussed in Zhi et al. [55]. In our study, we either used the recombination maps provided by Bhérer at al. [56] or set a flat recombination rate between each pair of sites. At this point we would also recommend to anybody seeking to put in place an ANONYMP style system, to take advantage of the fact that most imputation software contain a ‘K’ parameter which details how many reference haplotypes are to be considered for the forward-backward algorithm. This pre-selection of states can be performed by 3-Compare by simply inferring the hamming distance for each reference haplotype, simply by counting the number of discordant entries in the compare matrix in each column and retaining only the K best scoring columns. This would essentially represent the pre-selection of reference haplotypes used by IMPUTE2 [57] and it is what we have put in place in the demonstrative code that we provide with this work. In this way, the 4-PPM server is only ever working with a very small subset of the reference panel, setting K = 100 for example. Some precision in imputation could be lost at this point as more modern methods have more sophisticated ways of choosing the top K haplotypes. Once 4-PPM has ran the HMM, the posterior probabilities need to be linearly interpolated to positions not present in the target data, but present in the reference panel data; requiring some communication with 2-Reference. The HMM we implement is simply the Li-Stephens [37] mosaic copying model. We set the same flat error parameter of epsilon = 0.0001 for mismatches between observed and copying-state alleles as IMPUTE5 [38]. We also set the effective population size ($N_{e}$) to 10,000. It would likely be necessary to provide 4-PPM with a noised recombination map for the chunk in question to be imputed as in Zhi et al. [55]; but we leave that potential extension of ANONYMP aside in this work. We also envisage that some noise could also be added regarding the exact positions of the sites only seen in the reference panel within the chunk to be imputed without any great harm to the imputation; but again, that is not implemented in this proof-of-concept.

Once the forward-backward algorithm has completed, the final PPM matrix can then be completely reshuffled and noised by 4-PPM, and so need no longer be in matrix format, 2-Reference must do the same to their reference data (these steps are shown in the top-right corners of the boxes for 4-PPM and 2-Reference). At this point, via a careful co-ordination between both 4-PPM and 2-Reference, who send their shuffled and noised datafiles, including symmetrically inserted synthetic data points, to 5-Product who simply multiplies the two together. This would be a potential instance where homomorphic encryption could be employed but it would greatly increase the volume of data to transfer and would be largely equivalent in terms of privacy preservation to what we propose, since the current strategy already shares unstructured and shuffled vectors of random bits. The noise that is added here in ANONYMP is particularly complex in order to ensure that 1-User cannot infer information about the reference panel downstream. 2-reference adds random values to all of its genotypes in a manner so that for a given variant, it can predict what 1-User will calculate as a final dosage as being the sum of the true imputed dosage for 1-User’s data added to a predictable offset term. 2-Reference cannot know what the true dosages will be, but the offset term can be predicted, safely calculated, and sent to 1-User to facilitate the calculation of the final imputed dosage. This requires additional messages to be exchanged between 2-Reference and 4-PPM (to ensure symmetry in the inserted synthetic data points) as well as between 2-Reference and 1-User so that 2-Reference can provide 1-User with the offset term required to calculate the final dosage. 5-Product must therefore be separate from 4-PPM as otherwise 4-PPM would be able to see the data of 2-Reference. The data that 5-Product receives must remain unordered and noised so that 5-Product does not see the final imputed data of the target haplotypes. Finally, 5-Product passes their output back to 1-User, who (after exchanging messages with 2-Reference) can finally re-order and denoise their data, and then sum across columns (this represents the weighted sums of different copying states from the HMM) to finally attain imputed genotype dosages plus an offset coming from the addition of noise by 2-Reference and 4-PPM. The re-ordering of data that is performed by 1-User at this point will result in a matrix where the values in each row pertain to completely different reference panel haplotypes, and the values all include an additive noise term. This is of importance as otherwise 1-User could differentiate between reference and alternative alleles in the copying reference haplotypes as without the extra noise, the reference alleles would have a null contribution at this step which would be easily differentiable from alternate alleles.

Despite the measures of re-ordering different reference haplotype contributions and the extra noising steps added at this final stage of the calculation, the fact that 1-User is tasked with the summation of expected allele values across different reference haplotypes does mean that 1-User in fact receives more information under ANONYMP than they would in a typical imputation server paradigm. Though the extra information is rendered un-usable by the aforementioned protections, it is conceivable that a malicious actor in the place of 1-User could make a very high number of repeated imputation runs in an attempt to infer reference panel information [48]. To limit this risk, a deployed instance of ANONYMP could be configured to limit the number of runs from any given 1-User. To further avoid this risk in ANONYMP, we implement an alternative mode called ‘SecureSummation’ which includes a small modification to the protocol. Here a 6^th^ operator (6-Summation) is enlisted to perform just the summation step of the values coming from 5-Product; 1-User will still de-noise the final result after the summation. 6-Summation hence receives the majority of information that would otherwise go straight to 1-User from 4-PPM and 5-Product; as well as simply the number of SNPs involved in the imputation run from 1-User. 6-Summation completes the summation and sends the result onto 1-User. In the same way that the HMM steps of the imputation process needed to be done in as much isolation as possible (hence introducing the actor 4-PPM and heavily constraining what they can do), it is arguably necessary that this sensitive summation step be done in isolation of other tasks, hence in the SecureSummation mode, a 6^th^ operator is introduced, rather than giving this task to one of the other existing actors.

During the sum across columns (which in the default setting of ANONYMP is performed by 1-User, and by 6-Summation in the alternative SecureSummation mode), the noise that has been added combines together into a predictable offset which 1-User has received from 2-Reference and which simply needs to be subtracted to produce the final vector of imputed dosages. These final operations are shown in the top-right corner of the 1-User box (we do not include in Fig 3 the SecureSummation mode as it is essentially equivalent but with just some of the final operations taking place at 6-Summation and not at 1-User).

In this way, only 1-User gets to see the final output of the imputation, and the reference and target data are always manipulated in encrypted formats. What we are describing here are very simple encryptions (re-arranging data, adding noise, and adding synthetic data points) and hence all datafiles that need to manipulated can remain in plain text. More involved encryption such as discussed in the introduction could be used, but we aim here to put forward the possibility of avoiding such complexity.

**Fig 4:**
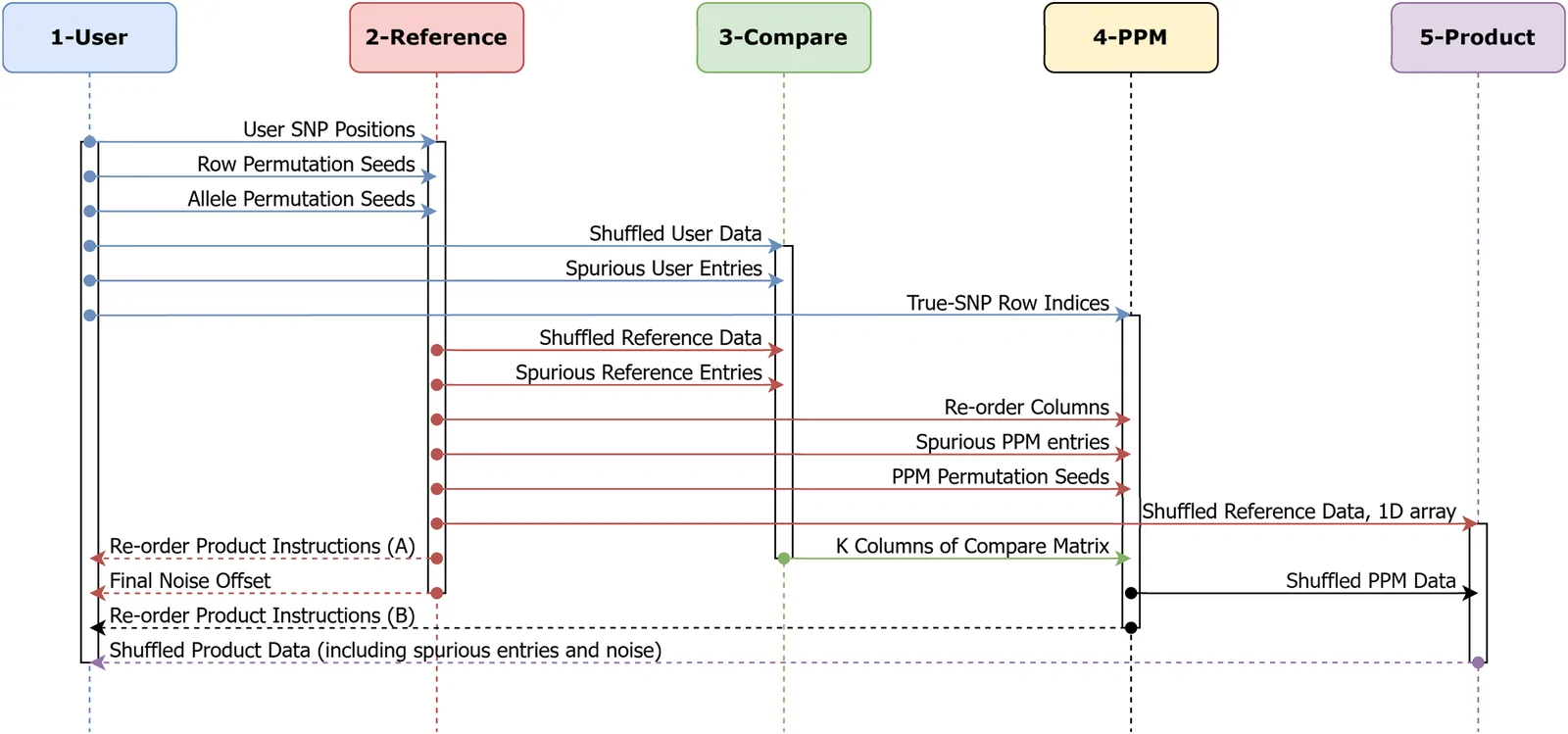
Flowchart summarising the communication between the 5 operators (details of which are given in Table 1).

The overall process requires many messages to be exchanged between operators; our current proof-of-concept implementation involves these messages simply being sent as R-object files. For a real-world application of ANONYMP, all these messages would have to be sent in a secure manner to avoid interception attacks. As an illustration of the additional measures to protect exchanged messages, ANONYMP can be employed with encrypted message transfers. All files can be encrypted prior to transfer and decrypted upon receipt using the OpenSSL command-line toolkit and password-based symmetric-key cryptography [58]. For a more in depth description of considerations on secure communications between operators, we point the reader to Bellafqira et al. [44]. In Table 1, we detail all the messages to be exchanged between operators in chronological order. A flowchart summarising the messages in Table 1 is given in Fig 4.

**Table 1:** Details of all messages exchanged between operators in the ANONYMP process.

	From	To	Message description	Message content labels
1	1-User	2-Reference	List of SNP array positions in 1-User’s data Instructions (random seeds) required by 2-Reference to apply the same re-arrangement of rows, and permutations of allele-status as were performed by 1-User on their data prior to 1-User sending their data to 3-Compare	• User SNP Positions • Row Permutation Seeds • Allele Permutation Seeds
2	1-User	3-Compare	The data of 1-User, with the shuffling of SNP order, no SNP information, permuted alleles, and additional spurious values in sufficient quantity to resize the list to a length common to all chunks	• Shuffled User Data • Spurious User Entries
3	1-User	4-PPM	The row indexes of the real SNPs in the list sent to 3-Compare	• True SNP Row Indices
4	2-Reference	1-User	Instruction on how to re-arrange the results that 1-User will eventually receive from 5-Product Offsets so that 1-User can remove the noise on their calculated imputed dosages that will be introduced by spurious PPM entries (see message 6)	• Re-order Product Instructions (A) • Final Noise Offset
5	2-Reference	3-Compare	The data of 2-Reference, with symmetric ‘encryptions’ as were added to 1-User’s data (message 2) and including spurious haplotypes and shuffled columns	• Shuffled Reference Data • Spurious Reference Entries
6	2-Reference	4-PPM	Instructions (random seeds) required by 4-PPM to revert the shuffling of columns provide to 3-Compare (Message 4) Spurious PPM columns to be inserted into the true PPM matrix as well as noise to add to the PPM matrix Instructions (random seeds) for shuffling all the values in the PPM matrix into a list or vector to be sent to 5-Product	• Re-order Columns • Spurious PPM entries • PPM Permutation Seeds
7	2-Reference	5-Product	The genotype values of the reference panel in a single dimension array, shuffled in a manner to be comparable to PPM values that will be sent to 5-Product (see message 10)	• Shuffled Reference Data (1D array)
8	3-Compare	4-PPM	A selection of columns from the comparison matrix	• K Columns of Compare Matrix
9	4-PPM	1-User	Further instructions (random seeds) on how to re-arrange the results that 1-User will eventually receive from 5-Product (to be combined with message 4)	• Re-order Product Instructions (B)
10	4-PPM	5-Product	The values of the PPM shuffled in a single dimension array	• Shuffled PPM Data
11	5-Product	1-User	The values of the PPM shuffled in a single dimension array multiplied by the equivalent list coming from 2-Reference (see message 7), with spurious values and noise (see message 6)	• Shuffled Product Data (including spurious entries and noise)

The ANONYMP imputation protocol framework and all source code are available at https://github.com/a-herzig/anonymp, with instructions for running tests on simulated datasets. The SecureSummation mode can be activate with the ‘-s’ flag, and encrypted message transfers can be activated with the ‘-m’ flag.

## Results

To test ANONYMP, we took simulated data presented in Herzig et al. [59] based on msprime [60] using the demographic scenario presented in Browning et al. [61]. Here we generated a reference panel of 45,000 individuals and a target dataset of 2,000 individuals to be imputed. Original code was developed to perform the ANONYMP imputation protocol on these simulated (see Data Availability Section). The five authors of the paper took on the roles of the five different operators (Figs 2 and 3) and were able to complete the imputation run; each working in a separate working environment. The results were verified as identical to an imputation run carried out directly, without the splitting of tasks and manipulations to data files.

**Fig 5:**
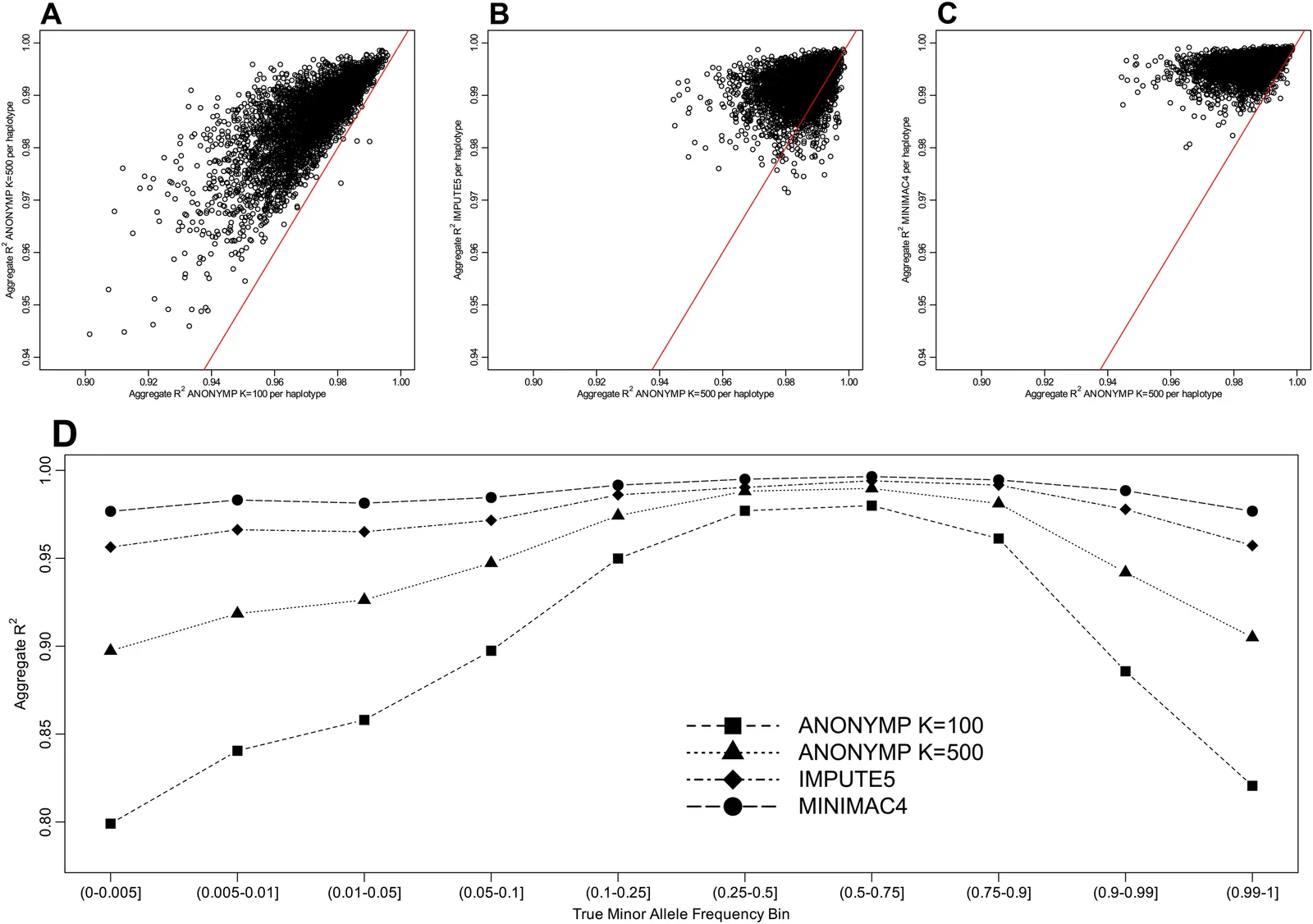
Comparison of imputation accuracy between ANONYMP, IMPUTE5 and MINIMAC4. In plots (a)-(c), squared correlations between imputed dosages and true allele are calculated across the 4,000 haplotypes of the 2,000 target individuals. We compare ANONYMP with two different settings: K = 100 or K = 500 where K describes the number of reference haplotypes used for the HMM. IMPUTE5 and MINIMAC4 were ran with default settings. In (d), aggregate correlations across all 2,000 individuals were calculated in different bins of minor (alternate in the reference panel) allele frequency.

Our simulated data only involved chromosome 15, which we split into 16 genomic regions or “chunks” using scripts provided with IMPUTE5 [62]. In Fig 5 we compare the imputation accuracy of ANONYMP to IMPUTE5 and MINIMAC4 [24]. Our HMM likely performed a little worse than leading imputation software as our model was notably rather less elaborate in terms of selecting the K reference haplotypes to use for imputation or the estimation of model parameters for the HMM. The importance of the K parameter is apparent in Fig 5(a) and 5(d). However, it was not our goal to develop a highly efficient of high-performance imputation software but rather to provide a basic rendition of the Li-Stephens model to demonstrate the possibilities for federation and anonymisation during the imputation process. The extra task involved in manipulating the different datafiles add relatively little time compared to the actual HMM calculations required for the imputation as can be seen when running the example imputation analyses as set out on the ANONYMP github. The transfers of data between operators do however greatly increase the total time required. The HMM naturally takes most of the total computation time though it should be stressed that our implementation of the Li-Stephens was rather naïve, being just for the purposes of this thought experiment, and hence is far slower that the heavily optimised imputation software IMPUTE5 and MINIMAC4.

Finally, we assessed the ability of 4-PPM to attempt to guess the series of alleles for a given target haplotype by assigning a reference allele when the row in the comparison matrix contains a majority of agreements and an alternative allele otherwise. On average over the 4,000 haplotypes, and for K = 100 and K = 500, this simple method guesses only 63.3% and 68.4% of the alleles correctly, respectively. This was not much better than simply guessing all alleles as being reference alleles which gave an average of 62.8% correct allele guesses. In Table 2, the processing time, memory usage, and file transfer size of the different steps of ANONYMP are detailed. The statistics relate to the imputation of a single individual in our simple proof-of-concept simulation. Different elements of the algorithm scale linearly depending on the number of target individuals, reference individual, the internal HMM parameter K, and the number of genomic regions to impute.

**Table 2:** Details of the computational burden of different sub-processes of ANONYMP. We were utilizing a Linux server and 16 cores and had up to 512GB of RAM available. For the approximate scaling-complexity reported, the following notation is used: T: number of target samples, C: number of “chunks” or genome regions to be imputed, N: number of reference individuals, K: number of HMM copying states.

Task Description	Approximate Processing Time	Computational Intensity and Scaling Complexity	Total File(s) Transfer Size
1-User data preparation and co-ordination with 2-Reference and 3-Compare	2 minutes	Low O(TCN)	1MB
2- Reference data preparation and co-ordination with all four other operators	30 minutes	Low O(CN)	2GB
3-Compare comparison matrix calculation and co-ordination with 4-PPM	5 minutes	Low O(TCN)	4MB
4-PPM forward-backward algorithm calculation	162 minutes	High O(TCK)	–
2-Reference creates additional spurious data entries and noise to add to the PPM matrix 4-PPM incorporates spurious data entries and noise from 2-Reference and sends out results to 5-Product and instructions to 1-User	103 minutes	Low O(TCK)	43GB
5-Product performs product calculation of PPM values and reference allele statuses; sends results to 1-User	25 minutes	Low O(TCK)	180MB
1-User re-organises the final results in order to construct imputed dosages	123 minutes	Low O(TCK)	–

## Discussion

In this work, we have laid out some novel ideas for the development of privacy-preserving imputation servers that go beyond previously presented methods. The key innovation is to split or federate different elements of the imputation algorithm between three distinct calculation nodes (3-Compare, 4-PPM, and 5-Product). Our work offers some alternative strategies to previous studies, focusing less on complicated data encryption and secure environments, but on decentralising the analyses and using multiple computation nodes to split different internal tasks of the imputation models. The key contribution of this study is this innovation for improving strategies and workflows for privacy-preserving imputation servers. Our approach could easily be combined with other previously presented measures for protecting sensitive data during genotype imputation. For example, key parts of the computation could be performed with homomorphic encryption: the comparison task of 3-Compare, the multiplication task of 5-Product, and the summation task of 6-Summation if applying the SecureSummation mode. These steps rely on simple arithmetic operations and so would be compatible with homomorphic encryption; the more computationally complex tasks of 4-PPM are far less suited to homomorphic encryption, hence why we have isolated this task and why previous methods have not achieved a fully homomorphically encrypted HMM application. But for the other more simple aforementioned tasks, the current ANONYMP implementation in R could be extended using the R package ‘homomorpheR’ [63] that employs the Paillier scheme [64]. However, this would greatly increase the size of data-transfers, which is already a weak point of our implementation. Furthermore, the decentralised approach of ANONYMP aims to avoid any need for such complicated encryption by splitting and isolating sensitive operations between different sites. Nevertheless, our framework would be completely compatible with homomorphic encryption if it were a pre-requisite for a real-world deployment of our protocol.

### Potential threats to ANONYMP

To explore the weaknesses of our strategy, we reasoned that the weakest link of ANONYMP would be at the 4-PPM server as here the variants within the genomic region to be imputed are put in order and genetic distances between adjacent cites may be given. 3-Compare and 5-Product have significantly less information. We showed that 4-PPM is unable to easily guess the target haplotype allele statuses (reference or alternative). But we should also consider the information that would be available for 4-PPM regarding the reference panel. In a given imputation run, the comparison matrix that has to be manipulated by 4-PPM resembles an MxK matrix of the K reference haplotypes selected by 3-Compare and the M variants present in both target and reference panels. The entries of the matrices are ‘true’/ ‘false’ values describing agreement between the target haplotype and each reference haplotype; information that might easily be stored as zeros and ones. This means that at this point 4-PPM essentially has a small portion of the haplotype reference panel; though without any point of reference regarding which rows or columns of the full haplotype reference panel it holds, or how the alleles are coded as here a zero is not necessarily aligned with a reference allele. Furthermore, this would allow 4-PPM to approximately calculate minor-allele frequencies and linkage disequilibrium between sites. Combining this with genetic distances between adjacent sites (even if the distances have added noise) could conceivably begin to provide 4-PPM with enough tools to infer which part of the genome it is analysing; particularly if it can cross-reference with knowledge of the sets of sites that are present on commonly used genotyping arrays. We would argue that the majority of this information could also be gleamed during the calculation of forward and backward probabilities by a malicious actor. This would strengthen the argument for developing a technique based on homomorphic encryption during the complex calculations of 4-PPM, but this is outside the scope of this current work. This would at least ensure greater assurance regarding the trustworthiness of the environment where the imputation algorithm takes place. But in all cases, all efforts would be quickly undermined by collusion between actors who are not supposed to share information. We thus maintain that splitting the HMM calculation across different locations adds sufficient protection. Table 3 details the vulnerabilities to individual level data that would be exposed by collusion between different pairs of operators. By collusion, we refer to pairs of operators sharing messages that they should not under the protocol of ANONYMP. For example, 2-Reference forwarding messages they received from 1-User on to 3-Compare.

**Table 3:** Potential ANONYMP data vulnerabilities arising from collusion between different pairs of operators.

Collusion risks	2-Reference	3-Compare	4-PPM	5-Product
1-User	Can reveal their data to each other	Can infer the reference panel data	Can infer the reference panel data	Can infer the reference panel data
2-Reference		Can infer the user’s data	Can infer the user’s data	Can infer the user’s data
3-Compare			Can infer array data from user and partial data (array positions only) from the reference panel but without genomic positions	Can likely infer reference panel data across many different imputation jobs
4-PPM				Can infer data (array and imputed variants) from user and the reference panel but without genomic positions

In Table 3, the capacities and limits for inference of genetic information of the 5 operators become apparent. ANONYMP is constructed so that 1-User never sees the reference panel data, even when summing across values coming from 5-Product where noise is added; but collusion with other actors would allow 1-User to unpick their results coming from 5-Product in order to construct sections of reference haplotype data. 2-Reference must not see data from 1-User but given than symmetric shuffling and noising are places on data from 1-User and 2-Reference, 2-Reference could uncover 1-User’s data if there is collusion between 2-Reference and one of the three other operators. They could effectively supply 1-User’s shuffled and noised data in a manner that 2-Reference could resolve it into the true data of 1-User. 3-Compare has noised and shuffled data from both 1-User and 2-Reference, and so clearly must not collude with either of those two operators, but nor with 4-PPM or 5-Product who have relevant information for 3-Product if they wished to derive the clear data of 1-User or 2-Reference. 4-PPM has a comparison matrix which bares similarities to the reference data, hence why they must have as little context as possible (chromosome and position information); they cannot directly get such information from collusion with 3-Compare and 5-Product but such collusion (particularly when repeated over many runs) would allow the colluding users to infer genotypes from 1-User and 2-Reference and via comparisons to public databases they could conceivably begin to reconstruct the full data of 1-User and 2-Reference by guessing context such as chromosome and position information.

At the sensitive 4-PPM stage, a few additional tricks could be added to aid in keeping this comparison matrix as uninformative as possible. By keeping K relatively low, leading to a small loss in accuracy, there would likely be many variants in full agreement across the K reference haplotypes with the target haplotype and these rows could be safely removed from the calculation by 3-Compare. There could also be variants in complete disagreement across the K reference haplotypes, which would correspond to rare alleles in the target haplotype. This eventuality is overall less likely but when occurring, again such rows could be removed (by 3-Compare) from the calculation with little impact. A more drastic approach would eventually be to drown out the possibility of 4-PPM making inference on the comparison matrices by simple asking it to perform more computation than necessary by mixing in additional synthetic imputation tasks and eventually even completely synthetic reference panels [49]. It would also probably be beneficial to split the chromosomes into sub-regions for imputation differently between different imputation runs; to avoid 4-PPM having the exact same dimensionality in the comparison matrices across all runs. As already discussed, at some point a more pragmatic approach would be to accept that during the HMM, either the input or output will have an unavoidable degree of similarity to sensitive individual level data. In this case, 4-PPM would best be hosted on a very secure computational environment, a relevant example being the Helmholtz imputation server [28] or the environments presented in Dokmai et al. [41].

An alternative protocol is in place in ANONYMP to address the question of equilibrium between the protection of the target and reference data. In the default mode, 1-User is tasked to perform a sum across different elements of the PPM matrix that have been multiplied by genotype values in the reference panel by 5-Product. We have placed additional complexity in terms of shuffling and noise at this stage to prevent 1-User from inferring information about the reference panel. However, a malicious user could use a strategy of repeatedly performing a large number of imputations using carefully constructed synthetic data as described in Mosca et al. [48] in an attempt to gain some information on the reference panel. Hence, allocating 1-User the task of this summation could open the door to some potential threats. There was thus an argument to make a modification to ANONYMP and to give this summation task to another operator in the ANONYMP framework. If 3-Compare, 4-PPM or 5-Product has this task, they would however have more information about the final results: the imputed genotypes of the user’s target data. The imputed genotypes of the target data would contain both rare genetic mutations as well as blocks of variants in tight LD (haplotypes) so that even if a very small portion of the imputed genome of the target individual were to be de-encrypted by a malicious operator, it would represent a far greater risk for re-identification attacks compares to the genotyping array data of the target individual that is already shared and manipulated as part of the imputation process. Hence this modification implies more weight on protecting the reference panel rather than the target data compared to the current implementation of ANONYMP. An appropriate compromise would be to simply ensure that different users can only perform a small number of imputation jobs to avoid threats to the reference panel data linked to this summation task in question. For an alternative application of ANONYMP, with a greater emphasis on protecting the reference panel data, we have implemented an alternate module where a 6^th^ operator is added to the protocol specifically to handle this summation step (see Methods). This does complicate an already intricate system, but would be a more appropriate solution if circumstances demand a greater insurance against the types of attacks described in Mosca et al. [48] against the reference panel. This would hence represent a shift in the equilibrium between protecting the reference panel and maximising the arguments for GDPR compliance with respect to protecting the user’s data by only performing upon it the strict minimum of sensitive operations at third-party locations.

### Limitations in applicability

Two further clear disadvantages of the ANONYMP framework are: firstly, the large number of messages and data-transfers that need to be made, and secondly, that leading publicly available imputation software cannot be used as they are.

The first consideration, we predict, could be overcome by changing slightly the schema proposed in Fig 3 so that instead of 2-Reference sending large files to 3-Compare each time, more simple instructions could be sent so that 3-Compare can re-arrange and re-noise previously received files from previous imputation jobs; similar recycling ideas could be easily applied in other moments in the protocol to avoid repetitive large data transfers. Thus, while the size of the messages to be sent to impute a single individual on chromosome 15 (see Table 2) appear excessive; we envisage that this could be easily reduced and co-ordinated in a way so that the size of messages for imputing many 100s of individuals would essentially be the same as for a single individual. Indeed, in the application of ANONYMP made available here, we specified that certain objects would always have a fixed size which both prevents certain operators guessing genomic locations and could facilitate such a recycling of previous messages of old imputation runs. In particular this would be relevant regarding the communication between 1-User and 2-Reference with 3-Compare, and the communications between 4-PPM and 5-Product and 2-Reference. These fixed sizes currently add restraints to the amount of spurious data entries that are to be added at different stages of ANONYMP (see Methods); though again this could be easily changed and tailored to different imputation server architectures.

For the second consideration, as we have shown, it is relatively simple to write a simple version of the Li-Stephens imputation algorithm, but this was both slower and more inaccurate than current software which have undergone years of development and optimisation. Hence, if a federated imputation server with an ANONYMP-style solution would greatly benefit from collaboration with current imputation software developers to isolate the different internal calculations and distribute them across different operators.

## Conclusions

We conclude with a description of what was the essential motivation behind the thought experiment that led to developing ANONYMP; and why for the moment it remains largely a thought experiment. The 1+M Genome program was established by the European Commission in 2018 with an objective to align whole-genome sequencing efforts across the EU to provide inference from over 1 million high quality genomes for clinical and research purposes. In October 2024, a sub-project began named Genome of Europe (https://genomeofeurope.eu/) with the objective of population-genetics analyses across 100,000 whole-genome sequences across 27 EU countries. One objective is to provide ancestry informed imputation haplotype reference panels with the caveat of each contributor to the project retaining their individual level WGS data within their own country. Analyses are to be federated between different calculation nodes as part of the Genomic Data Infrastructure (GDI) initiative. A plan is set out to collaborate with the recently opened Helmholtz imputation server where considerable work towards respecting GDPR considerations has been made. Given the multiplicity of potential calculation nodes via GDI, and the involvement of a secure third-party imputation environment in Munich, there could be the opportunity to deploy a federated imputation approach, in the style of ANONYMP. There will also be the important possibility to use such a set-up for more applications that imputation such as association testing [44] including association methods directly based on imputation algorithms [59].

Whether the simple encryptions involving data shuffling and noising proposed in ANONYMP will be sufficient to respect GDPR will likely depend on different national interpretations of the regulations. An in-depth discussion of data anonymisation for GDPR compliance is given in Ortega-Fernadez et al. [65]. It is hard to demonstrate full anonymisation of data to ensure GDPR compliance, particularly in areas such as human genetics where the list of potential external resources that could be used to re-identify individuals can be hard to anticipate (we could imagine that publicly available genomes, linkage-disequilibrium maps, recombination maps, phenotype information, records of haplotypes shared identical-by-descent could all be leveraged). Indeed, imputation servers have been shown to be implicitly vulnerable to re-identification of reference panel individuals [48]; this is because of the unavoidable problem of the purpose of imputation being to attempt to copy haplotypes from reference individuals to target individuals. Hence an imputation server that satisfies the strictest interpretations of GDPR data anonymisation may not be a realistic goal; but, as Ortega-Fernadez et al. [65] describe, the more realistic goal will be to find an optimum trade-off between utility and privacy and to demonstrate via a detailed analysis of re-identification risk that every realistic effort has been made to protect sensitive data whilst retaining a useful research application. Such a trade-off may seem like a worry for any attempt to attain GDPR compliance, but some pragmatism will always be required for issues surrounding the handling of human genomes given the wealth of possible analyses that can be performed that could lead to re-identifying individuals and breaking anonymisation; the previously cited work of Mosca et al. [48] being just one example. The key to achieving a workable legal basis for GDPR compliance will be to precisely define the purposes and the boundaries of an eventual imputation platform [32] along with exhaustive demonstrations of minimization of data vulnerability.

Until a specific context (with its own specific constraints and objectives) where a privacy-preserving imputation server involving federated calculation is decided upon, we felt that it is not possible to specifically develop and deploy a new tool or federated imputation pipeline. Hence in this work we have simply laid out one potential type of solution, which either on its own or more likely in conjunction with other methodology for privacy-preserving imputation servers, could be a useful innovation in the field. We have made available all code developed during this project to facilitate future methodological developments and discourse about privacy-preserving imputation software.
